# The influence of supportive leadership on hospitality employees’ green innovative work behavior: the mediating role of innovative climate and psychological empowerment

**DOI:** 10.3389/fpsyg.2025.1565408

**Published:** 2025-07-17

**Authors:** Arif Jameel, Noman Sahito, Wenjing Guo, Abid Hussain, Shahida Kanwel, Sania Khan

**Affiliations:** ^1^School of Business, Shandong Xiehe University, Jinan, China; ^2^Architecture and City Designing Department, College of Design and Built Environment King Fahd University of Petroleum and Minerals, Dhahran, Saudi Arabia; ^3^School of Nursing, Shandong Xiehe University, Jinan, China; ^4^School of Management, Jiangsu University, Zhenjiang, China; ^5^Department of Human Resource Management, College of Business Administration, Prince Sattam Bin Abdulaziz University, Al Kharj, Saudi Arabia

**Keywords:** green innovative work behavior, supportive leadership, psychological empowerment, innovative climate, hospitality and tourism, Saudi Arabia

## Abstract

**Introduction:**

Highlighting the implications of supportive leadership, the research examines the role of supportive leadership in predicting employee green and innovative work behaviors. The study also analyzes the mediating effect of innovative climate and psychological empowerment on the relationship between supportive leadership and green innovative work behavior.

**Methods:**

The data was gathered from full-time workers and managers employed at hotels in Saudi Arabia and analyzed using SPSS and AMOS. A study was conducted on a sample comprising 372 dyads of workers and their immediate supervisors.

**Results:**

The findings indicated that supportive leadership had a favorable correlation with green innovative work behavior, which was mediated by innovative climate and psychological empowerment among hotel employees. This study enhances the green innovative work behavior theory by identifying key psychological and organizational factors that motivate employees to engage in environmentally friendly activities within the hospitality industry.

**Conclusion:**

This research offers theoretical insights, practical applications, and suggestions for hospitality industry management.

## 1 Introduction

The hospitality sector has a significant impact on the environment and must implement proactive measures to mitigate this impact as consumers increasingly seek eco-friendly options (Cho and Yoo, 2021). The hospitality industry substantially impacts the degradation of the environment and “climate change” through its direct and indirect effects on natural resources and ecosystems. The hotel prioritizes environmental awareness for all stakeholders, especially addressing clients’ demands for ecological mindfulness, while endeavoring to uphold productivity and “sustainability” within the hospitality sector (Choudhary and Datta, 2024). The hotel sector incorporates the “green environmental” paradigm with creative practices to encourage “green innovative work behaviors (GIWB)” among personnel (Elkhwesky et al., 2022). The hospitality and tourism sectors are adopting new green service practices to minimize their environmental footprint, enhance social responsibility, and deliver value to consumers and stakeholders. Supportive leadership (SL), innovative climate (IC), and psychological empowerment (PE) are becoming more widely recognized as essential precursors to employees’ green innovative work behavior (GIWB), characterized by voluntary initiatives to cultivate and execute environmentally sustainable concepts and practices within the workplace (Jiang and Chen, 2021; Su et al., 2020). Supportive leadership arises when leaders actively offer encouragement, resources, and emotional support, cultivating a psychologically secure atmosphere that diminishes resistance to change and inspires people to participate in environmentally sustainable innovation (Zhao H. et al., 2024). This leadership style is crucial in fostering an innovative climate—a shared belief that creativity and experimentation, particularly in environmental sustainability, are valued and incentivized within the organization (Wang et al., 2022). The innovative climate indicates to employees that green projects are anticipated and endorsed, thereby encouraging proactive green innovative work behaviors. Psychological empowerment enhances these elements by fostering workers’ sense of competence, autonomy, and purpose in their employment, therefore reinforcing their intrinsic drive to engage in green innovations (Seibert et al., 2011). Supportive leadership and innovative climate collaboratively foster psychological empowerment, establishing a cohesive framework that enhances employees’ commitment to and involvement in GIWB (Li, 2022). Understanding the formation and interaction of these constructs is crucial for fostering sustainable innovation within organizations and enhancing environmental performance.

According to Kim and Lee (2013), GIWB in the service business refers to the creation and execution of new concepts for environmentally friendly services delivered to clients by service providers. GIWB enhances humanistic capital management, facilitating a competitive advantage by elevating entrance barriers for competitors, while leaders encourage workers to advocate for green innovative suppliers in the hospitality sector. This research examines green creative behavior within the hospitality sector, focusing on effective methods and initiatives facilitated by supportive leadership. Accountable leaders are the primary catalysts of employee innovation and satisfaction (Nguyen et al., 2023) while also being responsible for maintaining sustainable organizational settings (Elkhwesky et al., 2022). Existing research has demonstrated the fluctuation of various leadership styles in predicting workers’ environmental outcomes, including pro-environmental behavior (Afsar et al., 2020), green innovation, and organizational success (Bhutto et al., 2021). Supportive leadership techniques foster a constructive work environment, promote diversity and equality, and thereby enhance organizational performance (Dai et al., 2018).

Further research is needed to understand how a supportive leadership strategy encourages information sharing and promotes environmentally sustainable service practices in the hospitality industry. Green knowledge sharing promotes collaboration among hotel personnel, enabling them to integrate their talents and resources and fostering the development of novel services and activities aimed at mitigating environmental impacts within the hospitality sector (Rubel et al., 2023). However, research has not yet been conducted on the potential effect of supportive leadership on employee creative work behavior, which may facilitate knowledge sharing through the interchange of ideas (Rubel et al., 2023). “Social exchange theory” is associated with supportive leadership and innovative employee conduct. This theory, widely applied in “organizational behavior (OB)” and leadership literature, suggests that one’s self-perception and actions may be shaped by their group affiliations (Nguyen et al., consequently affecting their perceptions and interactions with colleagues in the workplace; Karatepe et al., 2020). The organizational services atmosphere has positively impacted an institute’s competitiveness, while workers’ green service innovations can mitigate the environmental impact of the hotel sector (Park and Min, 2020). The present study explores the role of supportive leadership in fostering creative green work behaviors and enhancing innovative climate and psychological empowerment among workers in the “hospitality” sector. This research investigates the correlation between “supportive leadership (SL)” and environmentally creative work behavior within the “hospitality” sector, emphasizing the influence of innovative climate through psychological empowerment. This research makes substantial contributions to the “hospitality literature” in several aspects. This research advances our comprehension of the determinants influencing GIWB among hotel workers by including SL as a pivotal feature. This study elucidates the beneficial effects of s by examining the effect of creative behavior among hotel staff.

**Figure 1:**
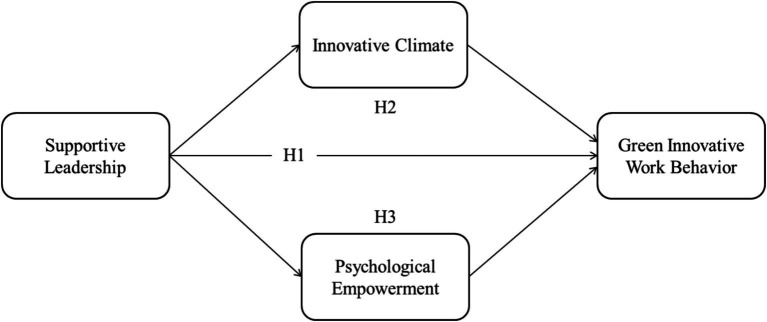
Theoretical model.

Supportive Leadership (SL), Innovative Climate (IC), and Psychological Empowerment (PE) are essential characteristics that collectively enhance the understanding of Green Innovation and Workplace Behaviors (GIWB). In this study, supportive leadership is theoretically enhanced by highlighting the leaders’ role in providing essential support, resources, and encouragement that inspire workers to participate in environmentally sustainable activities. An innovative climate facilitates an organizational environment that promotes innovation and transparency, thereby allowing for the formulation and execution of new green solutions. Simultaneously, psychological empowerment integrates human motivational theories with environmental behavior models by emphasizing how workers’ perceptions of autonomy, competence, and significance enable them to assume proactive roles in sustainability efforts. Practically, these constructs provide actionable insights for organizations: supportive leadership directs leadership development to foster supportive behaviors that promote green innovation; innovative climate aids in establishing workplace environments that facilitate experimentation and collaboration in sustainability initiatives; and psychological empowerment highlights the significance of empowering employees through autonomy and involvement, thus improving Engagement in environmentally friendly workplace practices. Supportive leadership, an innovative climate, and psychological empowerment collectively offer a robust framework that enhances both theoretical discussions and practical approaches in the dynamic domain of green, innovative work behaviors. Furthermore, our research contributes to the literature by clarifying the influence of innovative climate and psychological empowerment in amplifying the impact of supportive leadership on green innovative work behaviors among hotel employees. From a management perspective, the findings of this study will enhance workers’ awareness and Engagement in green initiatives, thereby fostering the development of new green services among hotel staff (see Figure 1).

## 2 Literature review and hypotheses development

### 2.1 Social Exchange Theory (SET)

Social Exchange Theory (SET) offers a significant theoretical framework for understanding the relationship between supportive leadership and workers’ green, innovative work behavior in the hospitality sector. Social Exchange Theory (SET) posits that social behavior stems from an exchange process in which individuals strive to maximize benefits and minimize costs in their relationships (Blau, 1964). In leadership, when managers demonstrate supportive behaviors—such as offering resources, encouragement, and recognition—employees regard these gestures as significant social exchanges. This perspective cultivates mutual attitudes and behaviors, encouraging employees to go beyond their formal positions and participate in voluntary green creative actions focused on environmental sustainability. Furthermore, supportive leadership fosters an innovative climate by cultivating an environment that promotes idea creation and experimentation, which workers reciprocate with heightened psychological empowerment. Empowered personnel possess the confidence and accountability necessary to foster green innovation, thereby fulfilling the exchange cycle anticipated by SET. Social Exchange Theory elucidates the postulated mediating processes by demonstrating how supportive leadership initiates a positive exchange relationship that fosters an innovative atmosphere and psychological empowerment, hence enhancing employees’ green creative work behavior.

### 2.2 Supportive leadership and green innovative work behavior

Recent research has examined the intricate link between supportive leadership (SL) and green innovative work behavior (GIWB), revealing the moderating and mediating processes involved. For instance, Gashema and Kadhafi (2020) examined the influence of psychological capital as a moderating variable. They found that elevated levels of “psychological capital” among workers correlated with a more significant effect of supportive leadership on green creative work behavior. This suggests that psychological capital enhances the positive impact of supportive leadership on green, innovative work behavior by serving as a valuable resource in social interactions with employers. Another study evaluated the mediational role of “intrinsic motivation (IM)” in the relationship between SL and GIWB (Demeško, 2017; Lee et al., 2020). The research’s findings indicated the substantial moderating effect of IM, implying that SLs promote employee engagement in GIWB by fulfilling their fundamental needs. These results align with the tenets of “Social Exchange Theory (SET),” which posits that individuals interact with others when they perceive the benefits outweigh the costs (Degutis et al., 2023). SET posits that individuals are more inclined to exhibit GIWB and respond positively to SL at the workplace when they perceive their boss acknowledges and values their contributions. Multiple research has investigated the intricate interactions between SL, GIWB, and SET (Knezović and Drkić, 2021; Prihandaka et al., 2022; Stein et al., 2020; Wang et al., 2022).

Additionally, Suifan et al. (2018) examined the role of perceived organizational support (POS) as a mediator in the link between GIWB and SL. Although some facets of employee creativity were deemed insignificant, POS emerged as a vital element. These findings suggest that supportive leaders may enhance GIWB by fostering a collaborative work environment that values and respects employee contributions. Significantly, Tan et al. (2021) examined the mediating functions of “perceived support for innovation” and innovation preparedness in the correlation between servant leadership (SL) and green innovative work behavior (GIWB). It was determined that “creativity and self-efficacy” acted as substantial mediators, with a more pronounced correlation noted among employees involved in greater social contacts. In conclusion, this research together indicates the interconnectedness of SL, GIWB, and SET. Leaders who foster a supportive work atmosphere, appreciate worker contributions, and incentivize innovation are more likely to motivate workers to participate in GIWB. Furthermore, the mediating role of KNS in the relationship between SL and GIWB has been emphasized within the tourism industry (Rafique et al., 2022). Employees are more likely to engage actively in GIWB when they believe their efforts are recognized and valued, thereby enhancing the beneficial effects of SL. Based on this review of the literature, we posit the subsequent hypothesis.

*H1*. Supportive leadership is positively associated with green innovative work behavior.

### 2.3 Supportive leadership and innovative climate

Recent research consistently indicates that supportive leadership is crucial for fostering an innovative environment within organizations. Supportive leaders who offer emotional support, resources, and autonomy foster psychological safety, enabling workers to take chances and express creative ideas without fear of adverse repercussions (Carmeli et al., 2013; Newman et al., 2017). This supportive atmosphere fosters intrinsic motivation and encourages information exchange, both of which are essential catalysts of innovation (Zhang and Bartol, 2010). Empirical research suggests that supportive leadership has a direct impact on perceptions of an innovative climate and an indirect influence on innovation outcomes by enhancing employees’ psychological empowerment and Engagement in creative processes (Frazier et al., 2017; Wang et al., 2022). Furthermore, in distant and hybrid work environments, it is essential to adopt supportive leadership behaviors to maintain innovative climates by addressing emerging issues related to communication and cooperation (Kniffin and Sapra, 2021). The recognized correlation between supportive leadership and a creative atmosphere suggests that leaders who prioritize support and facilitation are crucial for fostering organizational creativity. Based on this review of the literature, we posit the subsequent hypothesis.

*H2*. Supportive leadership is positively related to an Innovative climate.

### 2.4 Supportive leadership and psychological empowerment

Supportive leadership, characterized by trust, collaboration, and mutual support among team members, has been increasingly linked to psychological empowerment. Psychological empowerment, described as a multifaceted construct encompassing meaning, competence, self-determination, and efficacy (Spreitzer, 1995), has a profound impact on athletes’ motivation, performance, and overall well-being. Recent research indicates that supportive leaders demonstrating transformative behaviors’, including individualized assistance and the promotion of team autonomy, can augment athletes’ psychological empowerment by cultivating a feeling of competence and meaningful participation (Legutko, 2020). These leaders facilitate athlete empowerment by fostering intrinsic motivation and creating an environment conducive to personal growth, thereby enhancing their ability to govern their behaviors and decisions (Park, 2019). Furthermore, supportive leadership has been demonstrated to enhance collective efficacy and team cohesiveness, which are crucial for maintaining performance in competitive environments (Kim et al., 2021). The interplay between leadership styles and psychological empowerment has a profound impact on individual and team achievement, as empowered athletes exhibit increased dedication, ingenuity, and resilience in demanding situations (Al Otaibi et al., 2023). Supportive leadership is an essential technique for cultivating psychological empowerment, creating an atmosphere in which athletes feel valued and competent, hence improving both individual and team outcomes. Based on this review of the literature, we posit the subsequent hypothesis.

*H3*. Supportive leadership is positively related to Psychological empowerment.

### 2.5 Innovative climate and green innovative work behavior

An innovative organizational climate has been acknowledged as a crucial determinant of creativity and performance (Amabile et al., 1996). In the realm of environmental sustainability, an innovative climate is increasingly recognized as a pivotal catalyst for Green Innovative Work Behaviors, which encompasses employee actions that promote environmental conservation, including the formulation and implementation of eco-friendly practices (Ren et al., 2018). Recent studies highlight that a supportive and innovative environment, characterized by autonomy, risk-taking, and team cooperation, enhances employees’ propensity to engage in green and innovative work behavior (Zhang and Chin, 2024). Furthermore, studies highlight the importance of Green Human Resource Management (GHRM) methods, including training, performance assessments, and incentives, in promoting Green Innovative Work Behaviors by enhancing employees’ motivation and ability for green innovation (Jiang et al., 2023). The significance of leadership in fostering an innovative climate that aligns with environmental objectives has been underscored, with research indicating that transformational leadership has a favorable impact on workers’ environmental commitment and their subsequent green, innovative work behavior (Zhao and Zhang, 2024). Moreover, the alignment between personal beliefs and organizational environmental objectives, referred to as Person-Organization fit, has been shown to influence the correlation between organizational green activities and workers’ environmentally friendly behaviors’ (Chen and Zhang, 2024). These findings emphasize the need to foster an innovative atmosphere that promotes green and innovative work behavior, thereby improving organizational sustainability and environmental performance. Based on this review of the literature, we posit the subsequent hypothesis

*H4*. The innovative climate is positively related to Green innovative work behavior.

### 2.6 Psychological empowerment and green innovative work behavior

The concept of psychological empowerment refers to the internal motivation experienced by employees when they perceive themselves as competent, autonomous, and significant in their roles. It has four essential dimensions: meaning, competence, self-determination, and influence (Spreitzer, 1995). This notion has been thoroughly examined in the context of work behavior, with new research emphasizing its capacity to promote Green Innovative Work Behaviors. Green innovative work behavior refers to staff behaviors that foster environmental sustainability through innovative problem-solving and proactive measures (Anderson and Bateman, 2000). Empowered personnel are more likely to exhibit creative behaviors due to their increased sense of autonomy and confidence (Zhao D. et al., 2024). Studies have demonstrated that psychological empowerment can motivate workers to adopt environmentally sustainable behaviors and support organizational sustainability objectives (Kim et al., 2023).

Furthermore, leadership that fosters empowerment, particularly ethical and transformational leadership, has demonstrated the capacity to enhance the relationship between psychological empowerment and green innovative work behavior by aligning individual incentives with organizational sustainability objectives (Sarwar et al., 2024). Moreover, the incorporation of green human resource management strategies within organizations is seen as a crucial facilitator, ensuring that workers receive support in their endeavors to participate in sustainable innovations (Pham et al., 2024). These studies highlight the essential importance of psychological empowerment in creating an atmosphere that promotes green innovation, therefore enhancing employee well-being and organizational sustainability.

*H5*. Psychological empowerment is positively associated with green innovative work behavior.

### 2.7 Mediating role of innovation climate

Behavioral scholars frequently examine the association between leadership and employee creativity (Gupta and Singh, 2012; Jaiswal and Dhar, 2015; Zhang and Bartol, 2010; Zuraik and Kelly, 2018). Moreover, academics in the field of creativity have demonstrated an increasing interest in examining the association between supportive leadership and worker-green creativity (Gumusluoglu et al., 2017; Newman et al., 2020) have shown that supportive leaders have a significant influence on employee green innovation. Moreover, previous research has demonstrated that supportive leaders cultivate an “innovation climate (IC)” Jung et al. (2003) that influences the allocation of institutional resources and inspires personnel to pursue environmentally innovative results (Moghimi and Subramaniam, 2013). The interplay between contextual elements, namely supportive leadership and innovation climate, has prompted green innovation researchers to explore the indirect influence of supportive leadership on worker green creativity via innovation climate (Wang et al., 2017; Xue et al., 2022). The findings of this research are inconclusive. Gumusluoglu et al. (2017) found a negligible mediating influence of innovation climate on the relationship between supportive leadership and employee green creativity in their study of a Turkish sample.

Gumusluoglu et al. (2017) identified the mediating influence of innovation climate in a study including a Chinese sample. Nonetheless, in both investigations, Gumusluoglu et al. (2017) did not establish the “convergent validity” for the whole version of Scott and Bruce’s IC scale (Cavus and Bicer, rendering their findings questionable).

A new investigation by Ye et al. (2022) reveals that the relationship between SL and worker green creativity is mediated by the organization’s internal culture (IC). Cavus and Bicer (2016) found that workers’ perceptions of a favorable atmosphere, which facilitates easy access to institutional resources, moderate the impact of leadership on employees’ creative performance. The presence of such an environment at the group level provides an advantage to supportive leaders in enhancing juniors’ performance (Charbonnier-Voirin, 2011; Ye et al., 2022). An individual’s perspective of the innovation climate arises from the interactions between group members and their supervisor (Cavus and Bicer, 2016). The immediate boss serves as a prominent representative of the organization, leading subordinates to generalize their opinions of the supervisor to the organization as a whole (Cavus and Bicer, 2016). We posit that an innovative climate mediates the association between supportive leadership and employees’ green and innovative work behavior, as indicated by the aforementioned research.

*H6*. Group innovation climate mediates the association between supportive leadership and green innovative work behavior.

### 2.8 The mediating role of psychological empowerment

The concept of “psychological empowerment (PE)” originates from “industrial-organizational psychology” (Zimmerman, 1995). Empowerment denotes an individual’s capability to exercise “autonomy,” make decisions, take responsibility, and participate in corporate decision-making (Cattaneo and Chapman, 2010). The core premise of psychological empowerment is a psychological connection to an item. PE constitutes an element of positive organizational behaviors in the domain of psychological conduct. PE fosters optimism and the desire for achievement and success. It serves as a catalyst, inspiring individuals to attain superior performance levels (Almasradi et al., 2024). A previous study identified a correlation between PE and employee conduct (Li et al., 2018). They stated that PE significantly impacts employees’ in-role and additional-role activities. Previous research has demonstrated that SL affects the “psychological behavior” of employees (Li et al., 2018; Pacheco and Coello-Montecel, 2023). Leaders serve as intermediaries who seek to connect individuals and organizations, influencing employee behavior to enhance organizational success. Contemporary researchers have highlighted that organizational contexts—such as open information sharing, participatory decision-making, and decentralisation—are significantly associated with psychological empowerment (Llorente-Alonso et al., 2024; Saira et al., 2021). These enabling factors can be achieved through leadership. Supportive leadership fosters intrinsic task motivation among organizational members by promoting open communication and constructive debate (Simons, 2023; Zhang et al., 2023). Additionally, scholars have identified green innovative work behavior as a significant result of psychological empowerment (Afridi et al., 2023; Bhutto et al., 2021). Setyaningrum et al. (2023) demonstrate that elevated levels of green creativity are positively correlated with the perception of choice in one’s behaviors.

Empirical research on the relationship between supportive leadership, psychological empowerment, and innovative green behavior remains relatively insufficient (Fries et al., 2021; Zhang et al., 2023). Fries et al. (2021) contended that supportive leadership has a positive influence on green innovation through psychological empowerment. McCauley and Palus (2021) substantiated the theoretical assertion that supportive leadership impacts workers’ innovative behavior indirectly through positive employee experiences (PE). Jameel et al. (2023) observed that supportive leadership indirectly affects green creative behavior via the competence aspect of psychological empowerment. Moreover, additional research indicates that psychological empowerment serves as a vital mediating variable in the association between interactive supportive leadership and affective commitment as well as OCBE (Boedker and Chong, 2022; Jameel et al., 2023; Qing et al., 2020). Consequently, we anticipate that supportive leadership will encourage dialogue and discussion, create a collaborative and favorable environment for fostering a positive psychological experience (Simons, 2023; Zhang et al., 2016), and subsequently promote green innovative behaviors (Li et al., 2024; Peng et al., 2021). Therefore, we propose:

*H7*. Psychological empowerment mediates the association between supportive leadership and green innovative work behaviors.

## 3 Methodology

### 3.1 Sampling and data collection

The research employed a tripartite data-gathering methodology. For the objective of this research, 16 tourist hotels in Riyadh, Makkah, and Jeddah, Kingdom of Saudi Arabia, were first approached. This study employed stratified random sampling to ensure an accurate representation of the diverse range of hotels in Saudi Arabia within the sample. The hotel population is diverse, with considerable variations in size, star ratings, and consumer demographics. To address these discrepancies and improve the accuracy of the findings, the hotels were categorized based on these essential parameters. In doing so, we ensured that each subgroup—whether luxury hotels or budget hotels—was proportionately represented in the sample. This method not only reduces sampling error but also ensures that comparisons among various hotel categories are statistically significant and accurate.

Furthermore, stratified sampling enhances the efficiency of data gathering by reducing variability within each grouping, hence facilitating more precise findings. This strategy improves the generalizability of the study’s findings to the entire population of hotels in Saudi Arabia, ensuring that the results accurately represent the region’s unique hotel landscape. Consequently, stratified random sampling was a vital method for acquiring a representative and dependable sample, which is crucial for the integrity of the study’s results. A presentation was provided to the senior management of each hotel to outline the necessity and importance of the research and highlight its managerial consequences.

Representatives from 14 hotels expressed their readiness to engage in the survey. An orientation workshop for customer contact staff and their bosses was conducted at each participating hotel in coordination with the administration. Respondents were advised of the significance of their meticulous observation of each questionnaire item throughout the sessions. After each meeting, two separate kinds of sealed packages “(staff survey and supervisor survey),” each including a questionnaire, cover letter, and return envelope, were individually distributed. The participants were instructed to complete the questionnaires individually and submit them in a sealed package to their human resources office. The surveys distributed to customer contact personnel included items on supportive leadership, innovative climate, and psychological empowerment. At the same time, managers provided information about the green and creative work behavior of their subordinates. The customer contact employees included front desk staff, waitstaff, customer care personnel, and housekeeping staff, while department heads and team leaders represented supervisors. A total of 500 surveys were distributed to consumer contact staff, and 500 questionnaires were distributed to managers. The sample consisted of 372 customer contact workers and their direct bosses, resulting in a “response rate” of 74.4%. Out of the total, 46 supervisors completed the questionnaire about the green innovative work behaviors of 372 workers.

### 3.2 Measurement development

All research variables were assessed using a “five-point Likert scale,” where 1 indicated severe disagreement, and 5 indicated strong agreement. The questionnaires were composed initially in English, but they were then translated into Arabic by a native Arabic speaker. A different multilingual scholar translated the Arabic translation into English using the back-translation method (Schaffer and Riordan, 2003). Scholars evaluated the back-translation against the original “English version” and identified challenges in conceptual equivalence. The questionnaire items were derived from previous studies.

Supportive Leadership: Rafferty and Griffin (2004), with 3 “questions,” were employed to evaluate “supportive leadership”; an example question included “My supervisor considers my personal feelings when implementing actions that will affect me.”

Innovative Climate: We utilized the 16-item innovation climate measure by Scott and Bruce (1994) to evaluate the innovation climate. An example statement is, “Creativity is encouraged.” An individual’s impression of the innovation climate constitutes a collective mental model (Hofmann et al., 2003; Wang and Ma, 2013). Green Innovative Work Behavior: Supervisors utilized six items derived from the “green service innovative behavior” scale by Hu (2009) to evaluate their employees’ green innovative work behavior. Psychological Empowerment: The assessment of psychological empowerment utilized a 12-item, four-dimensional empowerment scale developed by Zimmerman. This scale is used extensively in recent research (Malik et al., 2021; Mathew and Nair, 2022). Illustrative inquiries for each of the four dimensions are: “My job activities are personally meaningful to me” (meaning or value), “I have significant autonomy in determining how I do my job” (self-determination or autonomy), “I have significant influence over what happens in my department” (impact or influence) and “I am self-assured about my capabilities to perform my work activities” (competency).

### 3.3 Common method bias (CMB)

Data collected concurrently from a single source may raise concerns about bias, casting substantial doubt on the study’s validity. The “Harman’s single-factor” test evaluated the issue of bias (Harman, 1976). The findings revealed that each feature of the proposed model can be classified into four categories, with the first component representing 39.78% of the variance. This figure signifies that existing biases are below 50%. Consequently, our data is free from any bias.

## 4 Statistical analysis and results

We utilized Analysis of Moment Structures (SEM) due to the nature of the data and the study’s objectives. Analysis of Moment Structures is well-suited for covariance-based structural equation modeling (CB-SEM), which enables the testing of complex relationships and the confirmation of theoretical models, with a focus on model fit and parameter estimates (Byrne, 2013; Collier, 2020). Since the study aims to test hypothesized causal relationships and evaluate the goodness-of-fit indices, AMOS was selected to ensure a more robust and statistically rigorous analysis. We adhered to the recommendations of Anderson and Gerbing (1988) regarding a two-step SEM approach, which begins with confirmatory analysis to verify model sufficiency. A proposed structural model was subsequently analyzed to evaluate the relationships among all variables. The fit indices utilized were “2/df, comparative fit index (CFI), standardized root mean square residual (SRMR), Tucker-Lewis index (TLI), and root mean square error of approximation (RMSEA).”

### 4.1 Descriptive statistics

Table 1 presents the mean and standard deviation (SD) for each variable, along with the correlations among the variables. Descriptive and correlation analyses reveal that SL is substantially correlated with innovative climate (*R* = 0.467, *p* < 0.01) and psychological empowerment (*r* = 0.458, *p* < 0.01). It exhibited a substantial correlation with green innovative work behavior (*R* = 0.487, *p* < 0.01). A considerable link existed between innovative climate and green innovative work behavior (*R* = 0.472, *p* < 0.01). A substantial association existed between PE and green innovative work behavior (*r* = 0.567, *p* < 0.01). Consequently, H1, 2, and 3 were first validated.

**Table 1:** Descriptive statistics.

Variables	Mean	Std.	1	2	3	4	5	6	7
AVE						0.872	0.776	0.782	0.675
Sex	1.23	0.39	-						
Age	2.35	1.06	−0.132*	-					
Edu	2.19	0.84	0.251**	0.137*	-				
SL	3.76	0.89	0.11*	−0.064	0.145*	-			
IC	3.45	0.93	−0.08	−0.085	0.117*	0.467**	-		
PE	3.87	0.85	−0.10	0.155*	0.086	0.458**	0.378**	-	
GIWB	3.92	0.88	0.18*	0.126*	0.234**	0.487**	0.472**	0.567**	-

*p < 0.05, **p < 0.01.

### 4.2 Measurement model assessment

In this study, the “measurement model” was verified using “Confirmatory Factor Analysis (CFA)” (Kline, 2023), and the “standard factor loadings, Cronbach’s alpha, and composite reliability” of every component are shown in Table 2 Supportive leadership, Innovative climate, Innovative climate, psychological empowerment, and Green innovative work behaviors have “alpha coefficients” of 0.92, 0.89, 0.96, and 0.90, in that order. These alphas are above the suggested value of 0.70 (Hair et al., 1998; Jameel et al., 2023). The “standardized loadings” for “Supportive leadership” ranged from 0.763 to 0.829, 0.730 to 0.895 for innovative climate, 0.767 to 0.893 for psychological empowerment, and 0.788 to 0.894 for green innovative work behavior. Each factor loading is above 0.50 [78] and makes a robust contribution. The “composite reliability (CR)” ranged from 0.87 to 0.93 for SL, innovative climate, PE, and green innovative work behavior, which is above the recommended value of 0.60 (Bagozzi et al., 1991; Jameel et al., 2024).

**Table 2:** Measurement model.

Factor	Items	Loadings	S.E.	T	C.R.	Α
SL	SL1	0.829	-	-	0.89	0.92
	SL2	0.763	0.054	16.92**		
	SL3	0.822	0.055	17.63**		
IC	IC1	0.799	-	-	0.87	0.89
	IC2	0.784	0.058	16.73**		
	IC3	0.894	0.054	17.51**		
	IC4	0.895	0.052	14.21**		
	IC5	0.813	0.049	17.62**		
	IC6	0.730	0.057	15.41**		
	IC7	0.736	0.058	15.53**		
	IC8	0.851	0.051	17.31**		
	IC9	0.840	0.048	16.73**		
	IC10	0.785	0.045	17.51**		
	IC11	0.867	0.049	16.21**		
	IC12	0.780	0.051	16.32**		
	IC13	0.764	0.050	14.61**		
	IC14	0.856	0.059	14.73**		
	IC15	0.795	0.058	17.51**		
	IC16	0.761	0.053	15.87**		
PE	PE1	0.828	-	-	0.93	0.96
	PE2	0.830	0.048	14.73**		
	PE3	0.784	0.046	15.61**		
	PE4	0.783	0.042	17.81**		
	PE5	0.884	0.048	16.42**		
	PE6	0.791	0.044	17.31**		
	PE7	0.893	0.049	15.73**		
	PE8	0.780	0.058	17.21**		
	PE9	0.767	0.059	15.63**		
	PE10	0.829	0.050	17.31**		
	PE11	0.841	0.051	15.55**		
	PE12	0.789	0.047	14.73**		
GIWB	GIWB1	0.872	-	-	0.88	0.90
	GIWB2	0.856	0.052	16.56**		
	GIWB3	0.894	0.054	17.25**		
	GIWB4	0.877	0.048	15.56**		
	GIWB5	0.846	0.056	17.18**		
	GIWB6	0.788	0.054	15.29**		

**p < 0.01.

Furthermore, we conducted a serial-wise confirmatory factor analysis to verify that the model accurately represented various components. The proposed four-factor model (SL, IC, PE, GIWB) demonstrated an adequate match to the data: χ^2^ = 1375.012, df = 225, CFI = 0.957, TLI = 0.963, RMSEA = 0.062, SRMR = 0.033 (Table 3). The proposed four-factor measurement model is the most suitable among all the others in Table 3.

**Table 3:** CFA results.

Factor model	χ2	Df	CFI	TLI	RMSEA	SRMR
Four-factor model: (SL, IC, PE, GIWB)	1375.012	225	0.957	0.963	0.062	0.033
Three-factor model: (SL, GIWB + IC, PE)	1577.213	247	0.887	0.896	0.123	0.114
Two-factor model: (SL + IC + GIWB, PE)	2142.765	226	0.784	0.678	0.135	0.132
Single factor model: (SL + IC + PE + GIWB)	2457.644	278	0.653	0.657	0.151	0.147

### 4.3 Hypotheses testing

The findings of this study corresponded with the methodology defined by Hayes (2017) 74 and Baron and Kenny (1986). Table 4 demonstrates a significant relationship between supportive leadership and green innovative work behaviors (*β* = 0.313, *p* < 0.001). It validated the H1 of our study. Baron and Kenny (1986) assert that the preliminary requirement for mediation is fulfilled. A substantial positive link was subsequently established between supportive leadership and innovative climate (*β* = 0.534, *p* < 0.001). A significant positive link was subsequently established between supportive leadership and psychological empowerment (*β* = 0.348, *p* < 0.001). Thus, the study’s results validated the second criterion of mediation and hypotheses H2 and H3. Innovative climate exhibits a strong correlation with green innovative work behavior (*β* = 0.342, *p* < 0.001), and psychological empowerment also shows a strong correlation with green innovative work behavior (*β* = 0.412, *p* < 0.001). These results validated the H4 and H5. Mediation was evaluated following the parameters established by Preacher and Hayes. Following the guidelines of Baron and Kenny (1986), the researchers assessed the significant indirect effects of bootstrapping the sample distribution. The results demonstrated that the indirect effect of supportive leadership on green innovative work behavior is substantial (*β* = 0.187, *p* < 0.001), (S.E. = 0.055), and (t = 3.338). The bootstrapping findings at a 95% confidence level for all confidence intervals did not encompass zero (Lower Level of Confidence Interval (LLCI) = 0.164, Upper Level of Confidence Interval (ULCI) = 0.273). The results also demonstrated that the indirect effect of supportive leadership on green innovative work behavior is substantial (*β* = 0.183, *p* < 0.001), (S. E. = 0.052), and (t = 3.519). The bootstrapping findings at a 95% confidence level for all confidence intervals did not encompass zero (Lower Level of Confidence Interval (LLCI) = 0.168, Upper Level of Confidence Interval (ULCI) = 0.278). Thus, these findings validate H6 and H7, as indicated in Table 4. The R^2^ values in the table represent the amount of variance in the dependent variables accounted for by the independent variables across different routes, with higher values indicating greater explanatory power. The path H5 has the highest R^2^ value of 0.30, indicating that PE accounts for 30% of the variance in GIWB. This is followed by H2, with an R^2^ of 0.25, which suggests that SL accounts for 25% of the variance in IC. The remaining routes, specifically H1, H3, and H4, exhibit R^2^ values ranging from 0.18 to 0.22, indicating considerable explanatory power. Cohen’s f^2^ values indicate the magnitude of the effect, with values of 0.02, 0.15, and 0.35 denoting small, medium, and significant effects, respectively. H5 has the most substantial effect size, with an f^2^ of 0.36, indicating a significant effect, while H2 and H4 have medium to moderate effect sizes, with f^2^ values of 0.33 and 0.27, respectively. The routes have medium to significant impacts, with H5 and H2 providing the most substantial contributions to the model.

**Table 4:** Hypothesized relationships (H1–H7).

Path		Β	S. E.	*t*-value	Bias-corrected 95% CI		*p*-value	R^2^	Cohen’s f^2^
					LLCI	ULCI			
Direct effects									
H1	SL → GIWB	0.313	0.060	5.233	0.361	0.471	<0.01	0.2	0.25
H2	SL → IC	0.534	0.077	6.961	0.242	0.455	<0.01	0.25	0.33
H3	SL → PE	0.348	0.059	5.898	0.230	0.383	<0.01	0.18	0.22
H4	IC → GIWB	0.342	0.053	6.452	0.245	0.386	<0.01	0.22	0.27
H5	PE → GIWB	0.412	0.071	5.802	0.365	0.459	<0.01	0.3	0.36
Indirect effect									
H6	SL → IC → GIWB	0.187	0.055	3.338	0.164	0.273	<0.01	0.22	0.25
H7	SL → PE → GIWB	0.183	0.052	3.519	0.168	0.278	<0.01	0.2	0.25

## 5 Discussion

The growing significance of green innovative work behaviors among employees in various corporate organizations has prompted academics to investigate the processes that enhance green creativity in the workforce. A persistent gap exists in the literature regarding the mediating effects on the prediction of green employee creativity through diverse antecedents. This study examines how supportive leadership, psychological empowerment, and an innovative atmosphere foster green and innovative work behaviors among employees, as well as the strength of this relationship. The study experimentally demonstrates that an employee’s assessment of their leader’s supportive leadership style, combined with their perceptions of their creative capabilities, significantly impacts their creative performance in a supportive innovation atmosphere, offering novel insights that contrast with previous studies. Our study findings aligned with the prior studies. Recent research by Wang et al. (2014) suggests that supportive leaders encourage green and innovative attitudes among their subordinate workers. The present research, building on the findings of Gupta and Singh (2015), identified a favorable correlation between supportive leadership and individual green creative behavior. The current study’s findings corroborate those of Gumusluoglu et al. (2017), offering empirical evidence that supportive leadership is more positively integrated within collectivist societies. Consequently, by exhibiting supportive leadership in a collaborative environment, leaders may deliver what their subordinates consistently desire: support, help, attention, and guidance (Alshebami and Seraj, 2022).

Furthermore, it has been demonstrated that “followers” with a “personalized relationship” with their bosses are more inclined to exhibit compliance and deference (ElMelegy et al., 2016). In a traditional collective context, such as Saudi Arabia, subordinates seek reliable, individualized connections with their superiors (Maspul, 2022). Moreover, the robust relationships between supportive leadership and both innovative climate and psychological empowerment substantiate, consistent with research highlighting the significance of leadership in influencing the work environment and augmenting employee motivation (Jameel et al., 2023; Wang et al., 2022). The substantial correlations between innovative climate and psychological empowerment with green innovative work behavior align with research indicating that empowered employees and an innovative environment are essential for fostering sustainable behaviors (Al-Ayed, 2024; Singh and Sarkar, 2012). The bootstrapping results confirm the indirect effects of supportive leadership on green and innovative work behavior, providing compelling evidence for the mediating roles of creative climate and psychological empowerment. These findings are consistent with Social Exchange Theory, which posits that supportive leadership cultivates a reciprocal exchange, leading workers who feel empowered and encouraged to engage in behaviors that benefit the organization, such as environmentally friendly and innovative work behavior.

A supportive leader fosters a robust, tailored relationship with each subordinate by recognizing their distinct needs through individualized consideration and attention. Supportive leaders not only wield organizational power akin to that of a superior, but they also serve as a benign resource upon whom subordinates may depend for individualized needs, such as instruction and coaching to enhance creative performance (Mittal and Dhar, 2016; Wang et al., 2014). Consequently, the study’s findings indicate that in Saudi Arabia, where the “leader-subordinate relationship” is characterized by deference and affection Bakhotmah, SL can elicit deference and affection from their subordinates, thereby facilitating enhanced creative performance. Furthermore, we identified a substantial mediation effect of innovative climate and PE on the association between SL and employee GIWB. The research indicates that supportive leaders can more successfully include their followers in environmentally creative activity when those followers perceive an organizational climate conducive to innovation. This confirms that individuals exhibit enhanced creativity under supportive leaders when they sense sufficient support for innovativeness regarding the availability of resources, incentives, and recognition. The absence of a favorable innovation atmosphere can adversely impact an individual’s perception of their creative abilities, regardless of their actual competence. The findings contribute to the literature on green innovation by highlighting the crucial role of leadership, empowerment, and climate in promoting sustainable organizational practices while also refining the theoretical framework of reciprocal interaction in the workplace.

### 5.1 Theoretical and managerial implications

This research makes a significant contribution to both theory and practice by examining the green innovative work behaviors (GIWB) of employees regarding supportive leadership, psychological empowerment, and an innovative atmosphere within the Saudi Arabian hospitality industry. This study is the first investigation into the correlation between supportive leadership and employee green innovative behavior, therefore augmenting previous theories through the incorporation of green innovation within the hospitality sector. The research highlights the essential role of supportive leadership in enhancing employee creativity and problem-solving by providing intellectual resources, emotional support, and stability. It underscores the mediating functions of psychological empowerment and innovation atmosphere in improving the impact of supportive leadership on green innovative work behaviors, indicating that employees’ creativity may be hindered in the absence of a robust sense of empowerment. These findings are particularly relevant to Saudi Arabia’s hospitality sector, offering actionable guidance for hotel managers to foster employee creativity through customized leadership methods, targeted training initiatives, and the creation of a nurturing and inventive workplace environment. This study provides a framework for Saudi hotels to enhance customer service, employee performance, and innovation, in line with the overarching objectives of Vision 2030 for economic diversification and sustainability. The company’s incentive system promotes and recognizes creative achievements while providing stability to its followers in the event of failure due to unconventional work methods. Therefore, hotel managers must possess a comprehensive awareness of the theoretical and practical link between supportive leadership and green creative behaviors. By adopting a supportive leadership style, they may effectively harness their staff’s creative abilities to derive unique solutions for regular challenges. The data concerning the mediating function of psychological empowerment and innovative climate is significant for at least two reasons. Initially, organizational initiatives aimed at fostering employee green innovation may be ineffective if an individual lacks a firm conviction in their creativity. Secondly, augmented psychological empowerment and an innovative climate serve as a catalyst for many predecessors of workers’ green creativity in work behaviors. This research provides a suitable strategy for managers seeking to optimize green creativity among their subordinates by fostering a supportive innovation climate and enhancing employee psychological empowerment through regular training and coaching. The “quality of service” provided by tourist hotels relies on customer-contact staff, and the study’s findings present important recommendations for implementing innovative strategies in their services. In tourist hotels, highly interactive service interactions occur between consumers and customer-contact staff. The inventiveness of customer interaction staff is crucial for improving customer loyalty and satisfaction (Calabrese et al., 2021).

Consequently, managers must recognize that their supportive leadership style has a profound influence on the creative performance of customer-facing staff. They must implement a tailored strategy for each subordinate, understanding their needs and perspectives to provide suitable resources and support. Hotel managers, by instilling trust in their juniors, may inspire a vision for innovative performance. They must deliver consistent training to their juniors, catalyzing the enhancement of their talents and fostering a creative approach to their job. The IC of tourist hotels has been assessed as relatively poor. Consequently, cultivating a brave and trustworthy environment is of paramount importance (Chien et al., 2021). Consequently, tourist hotels must establish a secure and supportive environment to motivate their personnel to engage in unconventional practices that enhance client value. The study’s findings suggest that a lack of psychological empowerment may diminish the influence of SL and an innovative atmosphere on predicting worker-green creativity. Consequently, although bosses may demonstrate “individualized consideration” and offer “intellectual stimulation,” they must furnish the essential components through “training and development” initiatives Yadav and Dhar (2021) to enhance their “creative potential” and self-efficacy in addressing day-to-day problems innovatively.

### 5.2 Limitations and future research

This research has several limitations that require consideration. The survey-based and cross-sectional approach hinders the creation of causal links among supervisors’ leadership styles, the innovation climate, and employee creativity, complicating the assessment of their mutual effect over time. A longitudinal design would yield more comprehensive insights into the causal dynamics and enduring impacts of leadership on creativity, providing more accuracy and excluding recall bias. Moreover, despite the use of stratified sampling to ensure a representative sample across various subgroups within the organization, limitations may persist regarding the effectiveness of the stratification in accurately reflecting the diversity of employee experiences across different departments, roles, and levels of seniority. Future research may refine the stratification process to provide a more equitable representation of essential subgroups, particularly in businesses characterized by complex systems. This research relied on self-reported data; therefore, future research could opt for a cohort study technique or gather information from other sources to minimize recall bias. Future research could also use supervisor ratings for GIWB and the time-lag design. The study missed to a certain extent to sufficiently consider cultural variations, which are essential for comprehending creativity. Future studies should incorporate cross-cultural comparative analyses to investigate the impact of individualistic and collectivistic cultural ideals on creativity across various contexts. The emphasis on the tourist lodging sector restricts the generalizability of the findings, indicating the need for replication in other industries, such as airlines, resorts, or travel services, to enhance the applicability of the results. Moreover, subsequent research should examine supplementary elements that drive creativity beyond leadership and the innovation milieu, as well as analyze the causes and consequences of inventive behavior within the hospitality industry. Mitigating these restrictions will provide a more comprehensive understanding of the factors influencing employee creativity, ultimately leading to more effective leadership practices and organizational strategies that promote innovation.

## 6 Conclusion

This research underscores the crucial importance of supportive leadership, psychological empowerment, and an environment that fosters creativity in promoting green innovative work behaviors (GIWB) among employees. The results confirm that supportive leadership is a crucial catalyst for green, innovative work behaviors, consistent with prior studies that emphasize the importance of leadership in promoting sustainable practices within organizations. This research highlights the substantial mediating roles of creative atmosphere and psychological empowerment in the relationship between supportive leadership and GIWB. Employees are more inclined to exhibit environmentally creative behaviors’ when they perceive empowerment and support within an organizational culture that prioritizes innovation. The study offers novel insights by examining the cultural context of Saudi Arabia, where the leader-subordinate dynamic is typically characterized by reverence and personalized attention. This cultural dimension enhances our understanding of the positive impact of supportive leadership on green innovation in collectivist countries. The study confirms that a lack of a conducive innovation environment may diminish employees’ perceptions of their creative potential despite possessing the necessary abilities. This research enhances the theoretical framework of reciprocal exchange in the workplace, providing significant empirical data about the influence of leadership, empowerment, and organizational climate on promoting green creative behaviors’. By highlighting these aspects, organizations can cultivate an environment that fosters creativity and sustainability, thereby enhancing both organizational performance and environmental well-being. In conclusion, the “dual mediation model” offers a comprehensive framework for understanding the impact of supportive leadership on green, innovative work behaviors. It emphasizes the intermediary functions of psychological empowerment and innovative climate, offering insights into how firms can foster a culture of environmental creativity and innovation through successful leadership.

## Data Availability

The raw data supporting the conclusions of this article will be made available by the authors, without undue reservation.

## Appendix

**Table:**

*Supportive Leadership (SL)*
My supervisor considers my personal feelings when implementing actions that will affect me
My supervisor shows concern for my well-being
My supervisor is approachable when I need to discuss work-related problems
*Innovative Climate (IC)*
Creativity is encouraged in this organization
New ideas are welcomed by management
Employees are rewarded for innovative thinking
Risk-taking is accepted as a necessary part of innovation
Management supports experimentation to improve work processes
Team members are encouraged to think outside the box
There is freedom to try out new ways of doing things.
The organization supports continuous learning and development
Suggestions for improvements are taken seriously by supervisors
Innovation is considered a priority in this workplace.
Collaboration and sharing of new ideas are encouraged.
Resources are available to support innovative projects
Mistakes made while trying new things are not punished
Employees have the autonomy to implement creative solutions
The organization adapts quickly to changes in the environment
There is a positive attitude toward change and innovation
*Green Innovative Work Behavior (GIWB)*
This employee often suggests new ideas to improve environmental sustainability at work
This employee actively implements environmentally friendly practices in their daily tasks
This employee modifies existing work methods to reduce environmental impact
This employee seeks out and applies new technologies or methods to promote green services.
This employee encourages coworkers to adopt environmentally responsible behaviors’
This employee takes initiative to solve environmental problems in the workplace
*Psychological Empowerment (PE)*
The work I do is meaningful to me
My job activities are personally important to me
I am confident about my ability to do my job well
There is freedom to try out new ways of doing things
I have mastered the skills necessary for my job
I feel capable of handling the responsibilities of my job
I have significant control over how I do my job
I can decide on my own how to carry out my work
I have autonomy in scheduling my work
My actions at work can influence important outcomes
I have a say in decisions that affect my work
I feel that I can make a difference in my organization

## References

[ref1] Afridi S. A. Shahjehan A. Zaheer S. Khan W. Gohar A. (2023). Bridging generative leadership and green creativity: unpacking the role of psychological green climate and green commitment in the hospitality industry. SAGE Open 13:21582440231185759. doi: 10.1177/21582440231185759

[ref9002] Afsar B. Bibi A. Umrani W. A. (2020). Ethical leadership and service innovative behaviour of hotel employees: the role of organisational identification and proactive personality. International Journal of Management Practice, 13, 503–520.

[ref2] Al Otaibi S. M. Amin M. Winterton J. Bolt E. E. T. Cafferkey K. (2023). The role of empowering leadership and psychological empowerment on nurses’ work engagement and affective commitment. Int. J. Organ. Anal. 31, 2536–2560. doi: 10.1108/IJOA-11-2022-3195

[ref3] Al-Ayed S. (2024). Green innovation influenced by employee innovative work behavior via moderating role of innovative leaderships. Cogent Bus. Manag. 11:2393741. doi: 10.1080/23311975.2024.2393741

[ref4] Almasradi R. B. Sarwar F. Droup I. (2024). Authentic leadership and socially responsible behavior: sequential mediation of psychological empowerment and psychological capital and moderating effect of perceived corporate social responsibility. Sustain. For. 16:6508. doi: 10.3390/su16156508

[ref9004] Alshebami A. S. Seraj A. H. A. (2022). Exploring the influence of potential entrepreneurs’ personality traits on small venture creation: The case of Saudi Arabia. Frontiers in Psychology, 13, 885980 35529569 10.3389/fpsyg.2022.885980 PMC9072652

[ref5] Amabile T. M. Conti R. Coon H. Lazenby J. Herron M. (1996). Assessing the work environment for creativity. Acad. Manag. J. 39, 1154–1184. doi: 10.5465/256995

[ref6] Anderson L. M. Bateman T. S. (2000). Individual environmental initiative: championing natural environmental issues in US business organizations. Acad. Manag. J. 43, 548–570. doi: 10.2307/1556372

[ref7] Anderson J. Gerbing D. (1988). Structural equation modelling in practice: a review and recommended two-step approach. Psychol. Bull. 103, 411–423. doi: 10.1037/0033-2909.103.3.411

[ref8] Bagozzi R. P. Yi Y. Phillips L. W. (1991). Assessing construct validity in organizational research. Admin. Sci. Q. 36, 421–458. doi: 10.2307/2393203

[ref9] Baron R. M. Kenny D. A. (1986). The moderator–mediator variable distinction in social psychological research: conceptual, strategic, and statistical considerations. J. Pers. Soc. Psychol. 51, 1173–1182. doi: 10.1037/0022-3514.51.6.1173, PMID: 3806354

[ref10] Bhutto T. A. Farooq R. Talwar S. Awan U. Dhir A. (2021). Green inclusive leadership and green creativity in the tourism and hospitality sector: serial mediation of green psychological climate and work engagement. J. Sustain. Tour. 29, 1716–1737. doi: 10.1080/09669582.2021.1902305

[ref11] Blau P. M. (1964). Justice in social exchange. Sociol. Inq. 34, 193–206. doi: 10.1111/j.1475-682X.1964.tb00843.x

[ref12] Boedker C. Chong K. M. (2022). The mediating role of accounting controls between supervisors' empowering leadership style and subordinates' creativity and goal productivity. Account. Finance 62, 4587–4614. doi: 10.1111/acfi.12795

[ref13] Byrne B. M. (2013). Structural equation modeling with Mplus: Basic concepts, applications, and programming. New York USA: Routledge.

[ref14] Calabrese A. Costa R. Ghiron N. L. Tiburzi L. Pedersen E. R. G. (2021). How sustainable-orientated service innovation strategies are contributing to the sustainable development goals. Technol. Forecast. Soc. Change 169:120816. doi: 10.1016/j.techfore.2021.120816

[ref15] Carmeli A. Gelbard R. Reiter-Palmon R. (2013). Leadership, creative problem-solving capacity, and creative performance: the importance of knowledge sharing. Hum. Resour. Manag. 52, 95–121. doi: 10.1002/hrm.21427

[ref16] Cattaneo L. B. Chapman A. R. (2010). The process of empowerment: a model for use in research and practice. Am. Psychol. 65, 646–659. doi: 10.1037/a0018854, PMID: 20873882

[ref17] Cavus M. F. Bicer M. (2016). Relationship between organizational ethical climate and innovative behavior: an example from Turkey. Int. J. Acad. Res. Bus. Soc. Sci. 6, 117–127. doi: 10.6007/IJARBSS/v6-i10/2347

[ref18] Charbonnier-Voirin A. (2011). The development and partial testing of the psychometric properties of a measurement scale of organizational agility. M@n@gement 14, 119–156. doi: 10.37725/mana.2011.0108

[ref19] Chen J. Zhang A. (2024). Greening the cubicle: unraveling the impact of corporate environmental ethics on employees’ green innovative behavior through the affective events theory. Curr. Psychol. 43, 25820–25835. doi: 10.1007/s12144-024-10950-3

[ref20] Chien S.-Y. Yang A. J.-F. Huang Y.-C. (2021). Hotel frontline service employees’ creativity and customer-oriented boundary-spanning behaviors: the effects of role stress and proactive personality. J. Hosp. Tour. Manag. 47, 422–430. doi: 10.1016/j.jhtm.2021.01.002

[ref21] Cho M. Yoo J. J.-E. (2021). Customer pressure and restaurant employee green creative behavior: serial mediation effects of restaurant ethical standards and employee green passion. Int. J. Contemp. Hospit. Manag. 33, 4505–4525. doi: 10.1108/IJCHM-12-2020-1087

[ref22] Choudhary P. Datta A. (2024). Bibliometric analysis and systematic review of green human resource management and hospitality employees' green creativity. TQM J. 36, 546–571. doi: 10.1108/TQM-01-2023-0044

[ref23] Collier J. (2020). Applied structural equation modeling using AMOS: Basic to advanced techniques. New York, USA: Routledge.

[ref24] Dai Y.-D. Hou Y.-H. Chen K.-Y. Zhuang W.-L. (2018). To help or not to help: antecedents of hotel employees’ organizational citizenship behavior. Int. J. Contemp. Hospit. Manag. 30, 1293–1313. doi: 10.1108/IJCHM-04-2017-0190

[ref25] Degutis M. Urbonavičius S. Hollebeek L. D. Anselmsson J. (2023). Consumers’ willingness to disclose their personal data in e-commerce: a reciprocity-based social exchange perspective. J. Retail. Consum. Serv. 74:103385. doi: 10.1016/j.jretconser.2023.103385

[ref26] Demeško N. (2017). Effects of transformational and transactional leadership styles on innovative work behavior: the role of employee's locus of control. Litvanya: ISM University of Management and Economics.

[ref27] Elkhwesky Z. Salem I. E. Ramkissoon H. Castañeda-García J.-A. (2022). A systematic and critical review of leadership styles in contemporary hospitality: a roadmap and a call for future research. Int. J. Contemp. Hosp. Manag. 34, 1925–1958. doi: 10.1108/IJCHM-01-2021-0074

[ref28] ElMelegy A. R. Mohiuddin Q. Boronico J. Maasher A. A. (2016). Fostering creativity in creative environments: an empirical study of Saudi architectural firms. Contemp. Manag. Res. 12:15015. doi: 10.7903/cmr.15015

[ref29] Frazier M. L. Fainshmidt S. Klinger R. L. Pezeshkan A. Vracheva V. (2017). Psychological safety: a meta-analytic review and extension. Pers. Psychol. 70, 113–165. doi: 10.1111/peps.12191

[ref30] Fries A. Kammerlander N. Leitterstorf M. (2021). Leadership styles and leadership behaviors in family firms: a systematic literature review. J. Fam. Bus. Strat. 12:100374. doi: 10.1016/j.jfbs.2020.100374

[ref31] Gashema B. Kadhafi M. I. (2020). Advancing employee’s innovative work behaviors in the workplace: the role of transformational leadership, positive psychological capital and effort-reward fairness. Bus. Rev. Soc. Sci. 2, 13–26.

[ref32] Gumusluoglu L. Karakitapoğlu-Aygün Z. Scandura T. A. (2017). A multilevel examination of benevolent leadership and innovative behavior in R&D contexts: a social identity approach. J. Leadersh. Organ. Stud. 24, 479–493. doi: 10.1177/1548051816648183

[ref33] Gupta V. Singh S. (2012). How leaders impact employee creativity: a study of Indian R&D laboratories. Manag. Res. Rev. 36, 66–88. doi: 10.1108/01409171211194834

[ref34] Gupta V. Singh S. (2015). Leadership and creative performance behaviors in R&D laboratories: examining the mediating role of justice perceptions. J. Leadersh. Organ. Stud. 22, 21–36. doi: 10.1177/1548051814535513

[ref35] Hair J. F. Black W. C. Babin B. J. Anderson R. E. Tatham R. L. (1998). Multivariate data analysis. Prentice Hall: New Jersey.

[ref36] Harman H. H. (1976). Modern factor analysis. Chicago: University of Chicago press.

[ref37] Hayes A. F. (2017). Introduction to mediation, moderation, and conditional process analysis: A regression-based approach. New York, USA: Guilford Publications.

[ref38] Hofmann D. A. Morgeson F. P. Gerras S. J. (2003). Climate as a moderator of the relationship between leader-member exchange and content specific citizenship: safety climate as an exemplar. J. Appl. Psychol. 88, 170–178. doi: 10.1037/0021-9010.88.1.170, PMID: 12675404

[ref39] Hu M.-L. M. (2009). Knowledge sharing and innovative service behavior relationship: Guanxi as mediator. Soc. Behav. Pers. 37, 977–992. doi: 10.2224/sbp.2009.37.7.977

[ref40] Jaiswal N. K. Dhar R. L. (2015). Transformational leadership, innovation climate, creative self-efficacy and employee creativity: a multilevel study. Int. J. Hosp. Manag. 51, 30–41. doi: 10.1016/j.ijhm.2015.07.008

[ref41] Jameel A. Ma Z. Li M. Hussain A. Asif M. Wang Y. (2024). The effects of social support and parental autonomy support on the mental well-being of university students: the mediating role of a parent–child relationship. Hum. Soc. Sci. Commun. 11, 1–8. doi: 10.1057/s41599-024-01688-3

[ref42] Jameel A. Ma Z. Liu P. Hussain A. Li M. Asif M. (2023). Driving sustainable change: the power of supportive leadership and organizational citizenship behavior in fostering environmental responsibility. Systems 11:474. doi: 10.3390/systems11090474

[ref43] Jiang Y. Asante D. Zhang J. Ampaw E. M. (2023). The influence of ambidextrous leadership on the employee innovative behavior: an empirical study based on Chinese manufacturing enterprises. Curr. Psychol. 42, 9452–9465. doi: 10.1007/s12144-023-04522-w

[ref44] Jiang D. Chen Z. (2021). Innovative enterprises development and employees’ knowledge sharing behavior in China: the role of leadership style. Front. Psychol. 12:747873. doi: 10.3389/fpsyg.2021.747873, PMID: 34744924 PMC8566712

[ref45] Jung D. I. Chow C. Wu A. (2003). The role of transformational leadership in enhancing organizational innovation: hypotheses and some preliminary findings. Leadersh. Q. 14, 525–544. doi: 10.1016/S1048-9843(03)00050-2

[ref46] Karatepe O. M. Aboramadan M. Dahleez K. A. (2020). Does climate for creativity mediate the impact of servant leadership on management innovation and innovative behavior in the hotel industry? Int. J. Contemp. Hospit. Manag. 32, 2497–2517. doi: 10.1108/IJCHM-04-2019-0423

[ref47] Kim K. Y. Atwater L. Jolly P. Ugwuanyi I. Baik K. Yu J. (2021). Supportive leadership and job performance: contributions of supportive climate, team-member exchange (TMX), and group-mean TMX. J. Bus. Res. 134, 661–674. doi: 10.1016/j.jbusres.2021.05.041

[ref48] Kim T. T. Lee G. (2013). Hospitality employee knowledge-sharing behaviors in the relationship between goal orientations and service innovative behavior. Int. J. Hosp. Manag. 34, 324–337. doi: 10.1016/j.ijhm.2013.02.005

[ref49] Kim K. Y. Messersmith J. G. Pieper J. R. Baik K. Fu S. (2023). High performance work systems and employee mental health: the roles of psychological empowerment, work role overload, and organizational identification. Hum. Resour. Manag. 62, 791–810. doi: 10.1002/hrm.22029

[ref50] Kline R. B. (2023). Principles and practice of structural equation modeling. New York, USA: Guilford Publications.

[ref51] Knezović E. Drkić A. (2021). Innovative work behavior in SMEs: the role of transformational leadership. Employ. Relat. 43, 398–415. doi: 10.1108/ER-06-2020-0262

[ref52] Kniffin L. E. Sapra S. (2021). Enhancing civic engagement through leadership education. J. Public Aff. 10:3. doi: 10.1080/22060708.2021.1914829

[ref53] Lee A. Legood A. Hughes D. Tian A. W. Newman A. Knight C. (2020). Leadership, creativity and innovation: a meta-analytic review. Eur. J. Work. Organ. Psychol. 29, 1–35. doi: 10.1080/1359432X.2019.1666355

[ref54] Legutko B. J. (2020). An exploration of authentic, servant, transactional, and transformational leadership styles in fortune 500 CEO letters. J. Leadersh. Stud. 14, 44–51. doi: 10.1002/jls.21651

[ref55] Li X. (2022). Green innovation behavior toward sustainable tourism development: a dual mediation model. Front. Psychol. 13:930973. doi: 10.3389/fpsyg.2022.930973, PMID: 35756259 PMC9226646

[ref56] Li X. Liu X. Huang Y. Li J. He J. Dai J. (2024). Evolutionary mechanism of green innovation behavior in construction enterprises: evidence from the construction industry. Eng. Constr. Archit. Manag. 31, 159–178. doi: 10.1108/ECAM-11-2022-0976

[ref57] Li H. Shi Y. Li Y. Xing Z. Wang S. Ying J.. (2018). Relationship between nurse psychological empowerment and job satisfaction: a systematic review and meta-analysis. J. Adv. Nurs. 74, 1264–1277. doi: 10.1111/jan.13516 29473198

[ref58] Llorente-Alonso M. García-Ael C. Topa G. (2024). A meta-analysis of psychological empowerment: antecedents, organizational outcomes, and moderating variables. Curr. Psychol. 43, 1759–1784. doi: 10.1007/s12144-023-10004-9

[ref59] Malik M. Sarwar S. Orr S. (2021). Agile practices and performance: examining the role of psychological empowerment. Int. J. Proj. Manag. 39, 10–20. doi: 10.1016/j.ijproman.2020.12.001

[ref60] Maspul K. A. (2022). Tourism development through creative economy in Saudi Arabia: sustaining coffee as a culinary destination in Buraidah. AJIRSS 1, 74–81.

[ref61] Mathew J. Nair S. (2022). Psychological empowerment and job satisfaction: a meta-analytic review. Vision 26, 431–440. doi: 10.1177/09722629221125529

[ref62] McCauley C. D. Palus C. J. (2021). Developing the theory and practice of leadership development: a relational view. Leadersh. Q. 32:101456. doi: 10.1016/j.leaqua.2021.101456

[ref63] Mittal S. Dhar R. L. (2016). Effect of green transformational leadership on green creativity: a study of tourist hotels. Tour. Manag. 57, 118–127. doi: 10.1016/j.tourman.2016.05.001

[ref64] Moghimi S. Subramaniam I. D. (2013). Employees' creative behavior: the role of organizational climate in Malaysian SMEs. Int. J. Bus. Manag. 8:1. doi: 10.5539/ijbm.v8n5p1

[ref65] Newman A. Donohue R. Eva N. (2017). Psychological safety: a systematic review of the literature. Hum. Resour. Manag. Rev. 27, 521–535. doi: 10.1016/j.hrmr.2016.07.002

[ref66] Newman A. Round H. Wang S. Mount M. (2020). Innovation climate: a systematic review of the literature and agenda for future research. J. Occup. Organ. Psychol. 93, 73–109. doi: 10.1111/joop.12264

[ref9001] Nguyen N. P. Hang N. T. T. Hiep N. Flynn O. (2023). Does transformational leadership influence organisational culture and organisational performance: Empirical evidence from an emerging country. IIMB Management Review, 35, 382–392.

[ref67] Pacheco P. O. Coello-Montecel D. (2023). Does psychological empowerment mediate the relationship between digital competencies and job performance? Comput. Human Behav. 140:107575. doi: 10.1016/j.chb.2023.107575

[ref68] Park C. K. (2019). The effects of transformational leadership on employee engagement: the role of Korean cultural values and psychological empowerment. Korean J. Manag. 32, 1671–1705.

[ref69] Park J. Min H. K. (2020). Turnover intention in the hospitality industry: a meta-analysis. Int. J. Hosp. Manag. 90:102599. doi: 10.1016/j.ijhm.2020.102599

[ref70] Peng H. Shen N. Ying H. Wang Q. (2021). Can environmental regulation directly promote green innovation behavior? Based on the situation of industrial agglomeration. J. Clean. Prod. 314:128044. doi: 10.1016/j.jclepro.2021.128044

[ref71] Pham V.-C. Wong W.-K. Bui X. T. (2024). Publication performance and trends in psychological capital research: a bibliometric analysis. J. Trade Sci. 12, 180–202. doi: 10.1016/j.jots.2024.03.001

[ref72] Prihandaka D. J. P. Rohman I. Z. Wijaya N. H. S. (2022). Supportive leadership and employee creativity: will leader-member exchange mediate the relationship. Ann. Manag. Organ. Res. 4, 35–45.

[ref73] Qing M. Asif M. Hussain A. Jameel A. (2020). Exploring the impact of ethical leadership on job satisfaction and organizational commitment in public sector organizations: the mediating role of psychological empowerment. Rev. Manag. Sci. 14, 1405–1432. doi: 10.1007/s11846-019-00348-3

[ref74] Rafferty A. E. Griffin M. A. (2004). Dimensions of transformational leadership: conceptual and empirical extensions. Leadersh. Q. 15, 329–354. doi: 10.1016/j.leaqua.2004.02.004

[ref75] Rafique M. A. Hou Y. Chudhery M. A. Z. Waheed M. Zia T. Chan F. (2022). Investigating the impact of pandemic job stress and transformational leadership on innovative work behavior: the mediating and moderating role of knowledge sharing. J. Innov. Knowl. 7:100214. doi: 10.1016/j.jik.2022.100214

[ref9005] Ren S. Xie Y. Zhu Y. Warner M. (2018). New generation employees’ preferences towards leadership style in China. Asia Pacific Business Review, 24, 437–458.

[ref76] Rubel M. R. B. Kee D. M.-H. Yusliza M. Y. Rimi N. N. (2023). Socially responsible HRM and hotel employees’ environmental performance: the mediating roles of green knowledge sharing and environmental commitment. Int. J. Contemp. Hosp. Manag. 35, 2645–2664. doi: 10.1108/IJCHM-04-2022-0394

[ref77] Saira S. Mansoor S. Ali M. (2021). Transformational leadership and employee outcomes: the mediating role of psychological empowerment. Leadersh. Organ. Dev. J. 42, 130–143. doi: 10.1108/LODJ-01-2020-0297

[ref78] Sarwar U. Aslam M. K. Khan S. A. Shenglin S. (2024). Optimizing human resource strategies: investigating the dynamics of high-performance practices, psychological empowerment, and responsible leadership in a moderated-mediation framework. Acta Psychol. 248:104385. doi: 10.1016/j.actpsy.2024.104385, PMID: 38968810

[ref79] Schaffer B. S. Riordan C. M. (2003). A review of cross-cultural methodologies for organizational research: a best-practices approach. Organ. Res. Methods 6, 169–215. doi: 10.1177/1094428103252966

[ref9003] Scott S. G. Bruce R. A. (1994). Determinants of innovative behavior: A path model of individual innovation in the workplace. Academy of management journal, 37, 580–6.

[ref80] Seibert S. E. Wang G. Courtright S. H. (2011). Antecedents and consequences of psychological and team empowerment in organizations: a meta-analytic review. J. Appl. Psychol. 96, 981–1003. doi: 10.1037/a0022676, PMID: 21443317

[ref81] Setyaningrum R. P. Kholid M. N. Susilo P. (2023). Sustainable SMEs performance and green competitive advantage: the role of green creativity, business independence and green IT empowerment. Sustain. For. 15:12096. doi: 10.3390/su151512096

[ref82] Simons E. (2023). The effect of empowering leadership and openness to experience on creativity, and the mediating role of engagement. Int. J. Organ. Innov. 13, 11–28.

[ref83] Singh M. Sarkar A. (2012). The relationship between psychological empowerment and innovative behavior. J. Pers. Psychol. 11, 215–222. doi: 10.1027/1866-5888/a000056

[ref84] Spreitzer G. M. (1995). Psychological empowerment in the workplace: dimensions, measurement, and validation. Acad. Manag. J. 38, 1442–1465. doi: 10.2307/256865

[ref85] Stein M. Vincent-Hoeper S. Gregersen S. (2020). Why busy leaders may have exhausted followers: a multilevel perspective on supportive leadership. Leadersh. Organ. Dev. J. 41, 829–845. doi: 10.1108/LODJ-05-2020-0289

[ref86] Su X. Xu A. Lin W. Chen Y. Liu S. Xu W. (2020). Environmental leadership, green innovation practices, environmental knowledge learning, and firm performance. SAGE Open 10:2158244020922909. doi: 10.1177/2158244020922909

[ref87] Suifan T. S. Abdallah A. B. Al Janini M. (2018). The impact of transformational leadership on employees’ creativity: the mediating role of perceived organizational support. Manag. Res. Rev. 41, 113–132. doi: 10.1108/MRR-07-2017-0207

[ref88] Tan A. B. Van Dun D. H. Wilderom C. P. (2021). Innovative work behavior in Singapore evoked by transformational leaders through innovation support and readiness. Creat. Innov. Manag. 30, 697–712. doi: 10.1111/caim.12412

[ref89] Wang Y. Chin T. Caputo F. Liu H. (2022). How supportive leadership promotes employee innovation under uncertainty: evidence from Chinese E-commerce industry. Sustain. For. 14:7491. doi: 10.3390/su14127491

[ref90] Wang R. Liu H. Jiang J. Song Y. (2017). Will materialism lead to happiness? A longitudinal analysis of the mediating role of psychological needs satisfaction. Pers. Individ. Diff. 105, 312–317. doi: 10.1016/j.paid.2016.09.008

[ref91] Wang G. Ma X. (2013). The effect of psychological climate for innovation on salespeople’s creativity and turnover intention. J. Pers. Sell. Sales Manag. 33, 373–387. doi: 10.1080/08853134.2013.798469

[ref92] Wang C.-J. Tsai H.-T. Tsai M.-T. (2014). Linking transformational leadership and employee creativity in the hospitality industry: the influences of creative role identity, creative self-efficacy, and job complexity. Tour. Manag. 40, 79–89. doi: 10.1016/j.tourman.2013.06.013

[ref93] Xue H. Luo Y. Luan Y. Wang N. (2022). A meta-analysis of leadership and intrinsic motivation: examining relative importance and moderators. Front. Psychol. 13:941161. doi: 10.3389/fpsyg.2022.941161, PMID: 36033069 PMC9413051

[ref94] Yadav A. Dhar R. L. (2021). Linking frontline hotel employees’ job crafting to service recovery performance: the roles of harmonious passion, promotion focus, hotel work experience, and gender. J. Hosp. Tour. Manag. 47, 485–495. doi: 10.1016/j.jhtm.2021.02.004

[ref95] Ye P. Liu L. Tan J. (2022). Creative leadership, innovation climate and innovation behavior: the moderating role of knowledge sharing in management. Eur. J. Innov. Manag. 25, 1092–1114. doi: 10.1108/EJIM-11-2020-0410

[ref96] Zhang X. Bartol K. M. (2010). Linking empowering leadership and employee creativity: the influence of psychological empowerment, intrinsic motivation, and creative process engagement. Acad. Manag. J. 53, 107–128. doi: 10.5465/amj.2010.48037109

[ref97] Zhang J. Chen Y. Liu J. (2016). Ethical leadership and OCBE: the influence of prosocial motivation and self-accountability. Acad. Manag. Proc. 2016, 1–10. doi: 10.5465/AMBPP.2016.145

[ref98] Zhang W. Chin T. (2024). How employee career sustainability affects innovative work behavior under digitalization. Sustain. For. 16:3541. doi: 10.3390/su16093541

[ref99] Zhang L. Kim D. Ding S. (2023). Cultivating organizational performance through the performance measurement systems: role of psychological empowerment and creativity. Front. Psychol. 14:1116617. doi: 10.3389/fpsyg.2023.1116617, PMID: 37034903 PMC10075081

[ref100] Zhao D. Cai W. Li Z. (2024). When differentiated empowering leadership improves team identification and individual work engagement: the combined roles of performance basis, team-member exchange, and employee self-efficacy. Curr. Psychol. 43, 36991–37008. doi: 10.1007/s12144-024-11315-4

[ref101] Zhao H. Chen Y. Zhao S. Wang B. (2024). Green inclusive leadership and hospitality employees’ green service innovative behavior in the Chinese hospitality context: the roles of basic psychological needs and employee traditionality. Int. J. Hosp. Manag. 123:103922. doi: 10.1016/j.ijhm.2023.103922

[ref102] Zhao W. Zhang Z. (2024). How CEO transformational leadership promotes firm innovation: mediating role of collective task self-efficacy. J. Organ. Change Manag. 37, 1633–1654. doi: 10.1108/JOCM-11-2023-0374

[ref103] Zimmerman M. A. (1995). Psychological empowerment: issues and illustrations. Am. J. Community Psychol. 23, 581–599. doi: 10.1007/BF02506983, PMID: 8851341

[ref104] Zuraik A. Kelly L. (2018). The role of CEO transformational leadership and innovation climate in exploration and exploitation. Eur. J. Innov. Manag. 21, 501–520. doi: 10.1108/EJIM-06-2018-0151

